# Screening of Onion (*Allium cepa* L.) Genotypes for Waterlogging Tolerance

**DOI:** 10.3389/fpls.2021.727262

**Published:** 2022-01-05

**Authors:** Pranjali A. Gedam, Dhananjay V. Shirsat, Thangasamy Arunachalam, Sourav Ghosh, Suresh J. Gawande, Vijay Mahajan, Amar Jeet Gupta, Major Singh

**Affiliations:** ICAR-Directorate of Onion and Garlic Research, Pune, India

**Keywords:** waterlogging, onion, genetic diversity, phenotyping, multivariate analysis

## Abstract

Onion production is severely affected by waterlogging conditions, which are created due to heavy rainfall. Hence, the identification of waterlogging-tolerant onion genotypes is crucial for increasing onion production. In the present study, 100 distinct onion genotypes were screened for waterlogging tolerance under artificial conditions by using the phenotypic approach in the monsoon season of 2017. Based on plant survival and recovery and changes in bulb weight, we identified 19 tolerant, 27 intermediate tolerant, and 54 highly sensitive onion genotypes. The tolerant genotypes exhibited higher plant survival and better recovery and bulb size, whereas sensitive genotypes exhibited higher plant mortality, poor recovery, and small bulb size under waterlogging conditions. Furthermore, a subset of 12 contrasting genotypes was selected for field trials during monsoon seasons 2018 and 2019. Results revealed that considerable variation in the morphological, physiological, and yield characteristics were observed across the genotypes under stress conditions. Waterlogging-tolerant genotypes, namely, Acc. 1666, Acc. 1622, W-355, W-208, KH-M-2, and RGP-5, exhibited higher plant height, leaf number, leaf area, leaf length, chlorophyll content, membrane stability index (MSI), pyruvic acid, antioxidant content, and bulb yield than sensitive genotypes under stress conditions. Furthermore, the principal component analysis biplot revealed a strong association of leaf number, leaf area, chlorophyll content, MSI, and bulb yield with tolerant genotypes under stress conditions. The study indicates that the waterlogging-tolerant onion genotypes with promising stress-adaptive traits can be used in plant breeding programs for developing waterlogging-tolerant onion varieties.

## Introduction

Onion (*Allium cepa* L.) is an important vegetable crop cultivated across 4.3 million hectares of land globally with a total production of 98 million tons (Food and Agriculture Organization of the United Nations, 2019). India is the second largest producer of onion after China, contributing approximately 20% of global production. More than a half of the onion production in India occurs in the Deccan Plateau. In this region, onion is cultivated as a rain-fed and irrigated crop during monsoon and post-monsoon seasons, respectively (Wakchaure et al., 2021). Climate change–induced frequent and prolonged waterlogging has been a severe constraint to onion production, particularly during the monsoon season (Gornall et al., 2010). Globally, flooding affects approximately 17 million km^2^ of land annually (Kaur et al., 2020). Estimation indicates that 14 million hectares of agricultural land in India are frequently flooded every year due to heavy rainfall (Kumar and Sharma, 2020). Onion is prone to excess moisture stress due to its shallow root system. Heavy rainfall periods during the bulb-development stage reduces bulb yield by 50–70% (Ghodke et al., 2018). Furthermore, the yield losses due to waterlogging vary with the rainfall intensity and its duration, the growth stage at which rainfall occurs, and sensitivity of genotypes for waterlogging (Pasley et al., 2020).

Waterlogging-induced oxygen depletion of soil is one of the major factors that affect plant growth and survival and causes high yield loss (Nishiuchi et al., 2012). Oxygen depletion adversely affects aerobic respiration in roots, which restricts energy formation, leading to a decline in net carbon assimilation and leaf chlorosis (Posso et al., 2018). This decline limits the plants’ water and nutrient absorption capacity and inhibits developmental processes, such as plant growth, photosynthesis, nutrient assimilation, dry matter accumulation, and crop yield (Garcia et al., 2020). Several agronomic practices have been developed to reduce onion yield loss under waterlogging conditions. However, the yield loss due to waterlogging accounts for about 50–70% during this season. Failure of monsoon season onion leads to a sudden rise in onion prices in the country during November and December. In addition, onion cultivars recommended for the monsoon season are generally susceptible to waterlogging stress.

A possible solution to overcome waterlogging stress is to identify the waterlogging-tolerant onion genotypes with promising adaptive traits to achieve the desired crop yield for flood-prone areas. Plants have evolved several adaptive changes in their phenotype, respiration, photosynthesis, endogenous phytohormone synthesis, and signaling cascade to respond to waterlogging stress (Zhou et al., 2020). Improved shoot and root growth with extensive aerenchyma formation and enhanced antioxidant activities to cope with reactive oxygen species are crucial for waterlogging-tolerant traits, which maintain the plant developmental process under waterlogged condition (Fukao et al., 2019). Therefore, identifying waterlogging-tolerant onion genotypes with promising adaptive traits is necessary to increase the yield in flood-prone areas. The phenotypic approach may act as a critical strategy for screening large and diverse germplasms to identify waterlogging stress–tolerant and sensitive genotypes. The stress-tolerance levels vary considerably across genotypes. Previous studies suggest that visual scoring of leaf chlorosis and grain yield under flood conditions are used for wheat genotype screening (Sundgren et al., 2018). In our previous study, drought-tolerant onion genotypes having adaptive traits were identified using drought stress indices and statistical analysis (Gedam et al., 2021), which indicated that several onion genotypes can accurately be screened for waterlogging tolerance based on their morphological, physiological, biochemical, and yield traits. Furthermore, principal component (PC) and multivariate analyses can be used for classifying the genotypes based on their genetic diversity and tolerance level under waterlogging stress. Therefore, the present study was conducted to evaluate the performance of 100 onion genotypes under artificially created waterlogging conditions and classify onion genotypes using PC and multivariate analyses. Furthermore, the performance of the identified contrasting genotypes was evaluated under excess moisture conditions to determine the onion genotype with waterlogging stress–tolerant traits under waterlogging.

## Materials and Methods

### Experimental Site

To evaluate onion genotypes for waterlogging tolerance, pot and field experiments were conducted at the research farm of ICAR-Directorate of Onion and Garlic Research (ICAR-DOGR), Pune, India (latitude N 18°84′, longitude E 73°88′, and 553.8 m above mean sea level), during the monsoon season (June–October) for 3 years (2017–2019). The climatic condition of the experimental site is characterized as a tropical dry humid climate with the mean annual maximum and minimum temperature of 27.6–32.8°C and 18.1–21.2°C, respectively (Supplementary Table 1). Mean annual rainfall of the experimental site is 821 mm, of which, 97–98% of the total precipitation is received during the southwest monsoon (June–October). The soil of the experimental site was clay loam soil (35% clay, 40% sand, and 20% silt) with a slightly alkaline pH (7.9).

### Experimental Material

A hundred diverse onion genotypes collected from the germplasm collection of ICAR-DOGR were used in the present study. In 2017, onion genotypes were screened for waterlogging stress tolerance under a waterlogged condition created artificially in a tank during monsoon season (June–October). Seedlings of onion genotypes were raised on the raised bed nursery. ICAR-DOGR–recommended fertilizer management practices, plant-protection measures, and intercultural operations were followed to obtain healthy seedlings.

### Experiment 1

For the screening experiment in monsoon season 2017, 45-day-old onion seedlings were transplanted in plastic containers (100 × 150 × 60 cm, height × length × width) of 24 kg capacity and filled with a 3:1 ratio of clay loam soil and farm yard manure. The experiment was laid out in a completely randomized design with three replications. Ten seedlings per container were transplanted for each genotype. The recommended doses of phosphorus, potassium, sulfur, and 22% of nitrogen were applied to the soil during transplanting, whereas the remaining 78% of nitrogen was applied in three equal parts at 15, 30, and 45 days after transplanting (DAT). The seedlings were raised under normal growing conditions up to 45 DAT. Waterlogging treatment was imposed by placing each container in a water tank at 45 DAT and maintaining the water level 3 cm above the soil surface. Plant survival was monitored every 24 h during the stress period. After 10 days of waterlogging, the containers were drained, and the plants were maintained in a stress-free environment until harvest. The number of recovered plants was recorded 10 days after relieving the stress. Furthermore, set of control plants were raised in plastic containers and maintained under a normal irrigation condition. The control plants with regular watering were protected from seasonal rainfall by using a rain-out shelter. The number and size of the bulbs formed were recorded at the time of harvest under the control and water-logged treatments. Based on the plant survival rate, recovery, and bulb weight, 12 contrasting genotypes were selected for field evaluation.

### Experiment 2

During monsoon seasons 2018 and 2019, the field experiments were performed to evaluate the 12 contrasting genotypes for waterlogging tolerance. For waterlogging treatment, seedlings were transplanted by maintaining the same spacing on a flat bed of 3 × 2 m. At the same time, the control plants were grown on a raised bed in a rain-out shelter. Forty-day-old onion seedlings were transplanted with a spacing of 10 cm between the plants and 15 cm between rows. Plants were watered regularly in controlled and waterlogging treatments until 45 DAT. Well-watered conditions were maintained in a rain-out shelter (control with 90% field capacity). In contrast, the waterlogged condition was maintained by using sprinkler and flood irrigation systems for 10 days continuously. The phenotypic and yield traits were recorded from both waterlogged and control plots. Plant samples were collected immediately after the stress period for analyzing the total chlorophyll, total phenol, and antioxidant activity. Furthermore, the samples collected at harvest were used for pyruvic acid and total soluble solids (TSS) estimation.

### Morphological Traits

In the field experiment, morphological traits, namely, plant height (PH), number of photosynthetically active leaves per plant (LN), leaf area (LA), leaf length (LL), shoot length (SL), and root length (RL), were assessed in waterlogged and control plots. These traits were also assessed during the recovery phase at 10 days after the stress period. The fourth fully matured leaf from the top of the plant was detached and used for measuring leaf area using the leaf area meter. Shoot and leaf length were measured and expressed in cm. Seedlings from each treatment were carefully uprooted and washed with water for measuring the RL. RL was measured and expressed in cm.

### Physiological Traits

#### Total Chlorophyll

Leaf chlorophyll content was determined spectrophotometrically (UV–Visible Spectrophotometer, Thermo Fisher Scientific, United States) by using the non-maceration method given by Hiscox and Israelstam (1979). The samples were prepared by taking leaf tissue (0.05 g) in a test tube containing 10 ml of dimethyl sulfoxide. The test tubes were incubated at 60°C for 60 min in a water bath. After cooling the test tubes at room temperature for 30 min, the absorbance was recorded at 645 and 663 nm. Total chlorophyll content was calculated using the formula given by Arnon (1949):


$$T\,o\,t\,a\,l\,\,c\,h\,l\,o\,r\,o\,p\,h\,y\,l\,l=\frac{\begin{array}{c} \left(20.2\times O\,D_{645}+8.02\times O\,D_{668}\right)\;\\ \times v\,o\,l\,u\,m\,e\,\,o\,f\,\,e\,x\,t\,r\,a\,c\,t\times W\,e\,i\,g\,h\,t\,\,o\,f\,\,s\,a\,m\,p\,l\,e\; \end{array}}{1000},$$

where OD_663_ is the absorbance at 663 nm and OD_645_ is the absorbance at 645 nm.

#### Membrane Stability Index

Membrane stability index (MSI) was measured at an interval of 5 days in control and waterlogged treatments to predict the level of cellular membrane injury (Sairam et al., 1997). The fourth leaf for each genotype was selected from both treatments and cut into 2 cm disks. Leaf disks weighing 100 mg were submerged in two sets of test tubes containing 10 ml of double-distilled water. One set of tubes were heated at 40°C for 30 min in a water bath, and electrical conductivity was measured on a conductivity bridge (C1). The second set was kept in a water bath for 10 min at 100°C, and electrical conductivity (C2) was recorded. MSI was quantified using the following equation:


$$M\,S\,I=\left[1-\frac{C\,1}{C\,2}\right]\times 100.$$

### Biochemical Traits

Leaf samples collected from the control and waterlogged plots were used for biochemical analyses.

#### Total Antioxidant Activity

Total antioxidant activity was estimated using ferric ion reducing antioxidant power (FRAP) assays (Benzie and Strain, 1996). The leaf extract was prepared in 80% aqueous methanol by macerating 1 g of fresh leaf tissue. A 150-μl aliquot from the leaf extract was mixed with 2850 μl of the FRAP reagent and incubated at room temperature for 30 min in the dark. The absorbance of ferrous tripyridyl triazine complex was determined at 593 nm by using a UV-visible spectrophotometer. Ascorbic acid was used as the standard. The FRAP values were expressed as mg ascorbic acid equivalents per g of sample fresh weight.

#### Total Phenol

Total phenol of the leaf samples was quantified spectrophotometrically by using the Folin Ciocalteu reagent (Pinelo et al., 2004). A fresh leaf sample (1 g) was homogenized in 10 ml of 80% aqueous methanol, and the homogenate was centrifuged at 5000 rpm for 10 min. Then, 200 μl of the extract was mixed with 1 ml Folin Ciocalteu reagent and incubated at room temperature for 5 min. After incubation, 800 μl of sodium carbonate was added, and the mixture was incubated for 2 h in the dark at room temperature. The absorbance was recorded at 765 nm using a UV-visible spectrophotometer. Gallic acid was used as the standard, and the results were expressed as gallic acid equivalent per gram fresh weight of the sample.

#### Pyruvic Acid

Pyruvic acid of the fresh onion bulb was estimated using the method given by Schwimmer and Weston (1961) using sodium pyruvate as the standard. The extract was prepared by homogenizing the 1 g core of the bulb sample in 1 ml double-distilled water and squeezed through muslin cloth. The extract was then allowed to settle at room temperature for 10 min, and 0.5 ml of the extract and 1.5 ml of 5% trichloroacetic acid were vortexed and then diluted to 20 ml with double-distilled water. The reaction mixture was prepared by mixing 1 ml extract, 1 ml double-distilled water, and 1 ml dinitrophenylhydrazine reagent incubated in a water bath at 37°C for 10 min. After incubation, 5 ml of 0.6 M sodium hydroxide was added to the reaction mixture, and the absorbance was recorded at 420 nm and expressed as micromole per gram fresh weight of onion bulbs.

#### Total Soluble Solids

Fresh onion bulbs were crushed, and the juices were extracted for evaluating the TSS by using a portable digital refractometer, and the values were expressed as ^0^Brix. The TSS of each treatment was quantified five times for each genotype, and the mean value was calculated.

### Analysis of Genetic Parameters

Number of Replications

The data obtained from 12 contrasting onion genotypes were analyzed for calculating the genotypic and phenotypic correlation by using the formula given by Kwon and Torrie (1964),


$$G\,e\,n\,e\,t\,i\,c\,\,V\,a\,r\,i\,a\,n\,c\,e\,\,\left(V\,g\right)=\frac{\begin{array}{c} G\,e\,n\,o\,t\,y\,p\,i\,c\,\,M\,e\,a\,n\,\,S\,q\,u\,a\,r\,e\;\\ -E\,r\,r\,o\,r\,\,M\,e\,a\,n\,\,S\,q\,u\,a\,r\,e\; \end{array}}{N\,u\,m\,b\,e\,r\,\,o\,f\,\,R\,e\,p\,l\,i\,c\,a\,t\,i\,o\,n\,s},$$

E⁢n⁢v⁢i⁢r⁢o⁢n⁢m⁢e⁢n⁢t⁢a⁢l⁢⁢V⁢a⁢r⁢i⁢a⁢n⁢c⁢e⁢⁢(V⁢e)⁢=E⁢r⁢r⁢o⁢r⁢⁢M⁢e⁢a⁢n⁢⁢S⁢q⁢u⁢a⁢r⁢e,

$$P\,h\,e\,n\,o\,t\,y\,p\,i\,c\,\,V\,a\,r\,i\,a\,n\,c\,e\,\,\left(V\,p\right)\,=\,\frac{\begin{array}{c} G\,e\,n\,e\,t\,i\,c\,\,V\,a\,r\,i\,a\,n\,c\,e\;\\ +E\,n\,v\,i\,r\,o\,n\,m\,e\,n\,t\,a\,l\,\,V\,a\,r\,i\,a\,n\,c\,e\; \end{array}}{N\,u\,m\,b\,e\,r\,\,o\,f\,\,R\,e\,p\,l\,i\,c\,a\,t\,i\,o\,n\,s}.$$

The genotypic coefficient and phenotypic coefficient variations were calculated using the following formula:


Geneticcoefficientofvariation(%)=VgX×100,

Phenotypiccoefficientofvariation(%)=VpX×100.

The heritability on entry mean basis were calculated by the following formula:


$$H\,e\,r\,i\,t\,a\,b\,i\,l\,i\,t\,y\,\,\left(H^{2}\right)=\frac{P\,h\,e\,n\,o\,t\,y\,p\,i\,c\,\,V\,a\,r\,i\,a\,n\,c\,e}{G\,e\,n\,e\,t\,i\,c\,\,V\,a\,r\,i\,a\,n\,c\,e}.$$

### Statistical Analysis

One-way analysis of variance was performed to analyze data generated from the pot experiments using SAS (Ver 9.3; SAS Institute, Cary, NC, United States). However, data from the field experiments were analyzed for three-way analysis of variance using year as a random factor and genotype and treatment as fixed factors to evaluate the impact of waterlogging stress on morphological, physiological, biochemical, and yield parameters of contrasting onion genotypes. The least significance difference (LSD) test at *p* = 0.05 was performed to determine the genotypic divergence and stress effect and to compare the phenotypic performance of tested genotypes for distinct traits under control and waterlogged conditions. The data of different traits recorded over 2 years were used for calculating Pearson’s correlation coefficient using SPSS software (Version 16.0). SPSS and XLSTAT software were used for performing PC and biplot analyses to determine the association among the genotypes based on different parameters.

## Results

### Experiment 1. Response of 100 Diverse Onion Genotypes to Waterlogging Stress

#### Assessing the Effect of Waterlogging Stress on Plant Survival, Stress Recovery, and Yield Traits

Plant survival, recovery percentage, and percentage change in the bulb weight of genotypes varied significantly under waterlogging stress (*p* < 0.0001) (Table 1). Of the genotypes exposed to waterlogging stress, 54 genotypes exhibited 100% survival, 24 genotypes exhibited 75–95% survival, and 17 genotypes exhibited 50–75% survival (Table 1). Furthermore, the genotypes Acc. 1656, Acc. 1624, Acc. 1617, Acc. 1640, and W-504 exhibited less than 50% survival with high plant mortality. During the recovery stage, six genotypes, Acc. 1615, Acc. 1622, Acc. 1661, Acc. 1666, Acc. 1708, and DOGR Hybrid-50, exhibited average plant growth with 100% plant recovery, and 10 genotypes had a plant recovery percentage of more than 85% (Table 2). The remaining genotypes exhibited less than 75% plant recovery. The bulb size and weight decreased significantly in all the genotypes under waterlogging stress (Table 3). Approximately 75 genotypes exhibited more than 80% bulb weight reduction due to waterlogging stress, whereas 14 genotypes exhibited 70–80% reduction in bulb weight. Of the 100 genotypes, Acc. 1666 exhibited 36.2% bulb weight reduction, and in about 10 genotypes, a 50–70% change in bulb weight was recorded under waterlogging stress.

**TABLE 1:** Genotypes grouped based on plant survival percentage under waterlogging stress.

Genotypes	Survival (95–100%)	Genotypes	Survival (95–100%)	Genotypes	Survival (75–95%)	Genotypes	Survival (50–75%)	Genotypes	Survival (<50%)
					
Tolerant		Relatively tolerant		Relatively sensitive		Sensitive			
Acc. 1133	100	Acc. 1627	100	Acc. 1604	88	Acc. 1606	71	Acc. 1617	50
Acc. 1608	100	Acc. 1635	100	Acc. 1616	90	Acc. 1613	75	Acc. 1624	44
Acc. 1609	100	Acc. 1636	100	Acc. 1618	90	Acc. 1621	75	Acc. 1640	50
Acc. 1615	100	Acc. 1638	100	Acc. 1620	90	Acc. 1633	56	Acc. 1656	40
Acc. 1619	100	Acc. 1639	100	Acc. 1628	89	Acc. 1645	63	W-504	50
Acc. 1622	100	Acc. 1649	100	Acc. 1629	80	Acc. 1660	70		
Acc. 1623	100	Acc. 1651	100	Acc. 1630	80	Acc. 1668	63		
Acc. 1625	100	Acc. 1653	100	Acc. 1632	80	Acc. 1701	70		
Acc. 1626	100	Acc. 1658	100	Acc. 1637	90	Acc. 1703	71		
DOGR Hy-1	100	Acc. 1661	100	Acc. 1644	88	Acc. 1704	70		
DOGR Hy-50	100	Acc. 1664	100	Acc. 1663	80	Acc. 1709	70		
DOGR Hy-56	100	Acc. 1666	100	Acc. 1696	90	Acc. 1712	70		
DOGR Hy-7	100	Acc. 1667	100	Acc. 1702	90	Acc. 1714	60		
W-043	100	Acc. 1697	100	Acc. 1706	88	B. Kiran	67		
W-085	100	Acc. 1708	100	B. Dark Red	90	DOGR Hy-2	67		
W-172	100	B. Raj	100	B. Shakti	90	DOGR Hy-4	70		
W-208	100	B. Red	100	B. Shweta	90	KH-M-4	70		
W-302	100	B. Safed	100	DOGR Hy-3	90				
W-306	100	B. Shubhra	100	DOGR Hy-6	90				
W-361	100	B. Super	100	DOGR Hy-8	78				
W-395	100	KH-M-1	100	KH-M-3	86				
W-396	100	KH-M-2	100	RGP-4	90				
W-408	100	Phule Safed	100	W-344	94				
W-448	100	RGP-1	100	W-355	90				
W-453	100	RGP-2	100						
W-507	100	RGP-3	100						
W-517	100	RGP-5	100						

**TABLE 2:** Genotypes grouped based on plant waterlogging stress recovery percentage.

Genotypes	Recovery (>80%)	Genotypes	Recovery (60–80%)	Genotypes	Recovery (60–80%)	Genotypes	Recovery (30–60%)	Genotypes	Recovery (30–60%)	Genotypes	Recovery (30–60%)	Genotypes	Recovery (<30%)
			
Tolerant		Relatively tolerant					Relatively sensitive					Sensitive	
Acc. 1133	90	Acc. 1604	76	B. Kiran	67	Acc. 1606	43	Acc. 1651	56	DOGR Hy-7	60	Acc. 1617	30
Acc. 1615	100	Acc. 1608	63	B. Red	78	Acc. 1609	50	Acc. 1656	40	DOGR Hy-8	56	Acc. 1625	20
Acc. 1619	90	Acc. 1613	63	B. Shubhra	67	Acc. 1618	60	Acc. 1658	56	KH-M-4	60	Acc. 1645	25
Acc. 1622	100	Acc. 1616	70	B. Shweta	70	Acc. 1621	50	Acc. 1660	50	Phule Safed	50	B. Dark Red	30
Acc. 1653	90	Acc. 1620	80	B. Super	80	Acc. 1623	50	Acc. 1668	50	RGP-1	60	W-344	22
Acc. 1661	100	Acc. 1626	70	DOGR Hy-56	63	Acc. 1624	33	Acc. 1696	50	RGP-2	50	W-504	25
Acc. 1664	89	Acc. 1627	70	DOGR Hy-6	70	Acc. 1629	40	Acc. 1702	50	W-085	50		
Acc. 1666	100	Acc. 1628	78	KH-M-1	80	Acc. 1630	60	Acc. 1709	40	W-306	50		
Acc. 1706	88	Acc. 1635	63	KH-M-2	67	Acc. 1632	40	Acc. 1712	50	W-361	47		
Acc. 1708	100	Acc. 1636	71	KH-M-3	71	Acc. 1633	33	Acc. 1714	60	W-396	57		
B. Shakti	90	Acc. 1644	63	RGP-3	80	Acc. 1637	60	B. Raj	50	W-408	56		
DOGR Hy-3	90	Acc. 1663	80	RGP-4	70	Acc. 1638	50	B. Safed	50	W-448	47		
DOGR Hy-50	100	Acc. 1667	70	W-043	80	Acc. 1639	60	DOGR Hy-1	60	W-453	60		
RGP-5	90	Acc. 1697	70	W-208	70	Acc. 1640	40	DOGR Hy-2	56	W-507	50		
W-172	89	Acc. 1701	70	W-302	70	Acc. 1649	40	DOGR Hy-4	60				
W-395	86	Acc. 1703	71	W-355	70								
Acc. 1133	90	Acc. 1704	70	W-517	67								

**TABLE 3:** Genotypes grouped based on percent change in bulb yield under waterlogging stress.

Genotypes	% Change in bulb weight (>90%)	Genotypes	% Change in bulb weight (>90%)	Genotypes	% Change in bulb weight (75–90%)	Genotypes	% Change in bulb weight (75–90%)	Genotypes	% Change in bulb weight (<75%)
						
	Sensitive				Relatively sensitive			Tolerant	
RGP-1	91.13	Acc. 1712	93.79	Acc. 1604	89.59	Acc. 1668	89.48	Acc. 1666	36.19
Acc. 1609	93.98	B. Dark Red	95.5	Acc. 1606	77.23	Acc. 1697	89.15	Acc. 1133	74.64
Acc. 1616	91.79	B. Kiran	96.17	Acc. 1608	86.82	Acc. 1701	83.38	Acc. 1615	62.44
Acc. 1617	93.46	B. Safed	93.1	Acc. 1613	83.99	Acc. 1704	84.33	Acc. 1619	66.77
Acc. 1621	92.69	B. Shakti	97.01	Acc. 1618	86.75	Acc. 1714	88.59	Acc. 1622	74.58
Acc. 1623	93.48	B. Shweta	91.3	Acc. 1620	84.41	B. Raj	87.23	Acc. 1661	63.51
Acc. 1624	95.73	B. Super	96.66	Acc. 1626	89.82	B. Red	89.4	Acc. 1703	75.73
Acc. 1625	92.19	DOGR Hy-1	95.85	Acc. 1627	90.02	B. Shubhra	89.29	Acc. 1706	75.38
Acc. 1632	94.92	DOGR Hy-4	93.25	Acc. 1628	88.05	DOGR Hy-2	90.65	Acc. 1708	62.96
Acc. 1633	95.1	DOGR Hy-56	93.15	Acc. 1629	85.59	DOGR Hy-6	80.78	DOGR Hy-3	71.81
Acc. 1635	91.16	DOGR Hy-7	94.81	Acc. 1630	76.93	KH-M-3	83.06	DOGR Hy-50	61.36
Acc. 1640	94.86	DOGR Hy-8	95.85	Acc. 1636	83.62	KH-M-4	88.17	KH-M-2	70.98
Acc. 1645	93.67	KH-M-1	96.06	Acc. 1637	86.81	Phule Safed	89.62	RGP-5	57.31
Acc. 1649	91.51	RGP-2	91.33	Acc. 1638	90.17	RGP-4	89.87	W-043	63.64
Acc. 1656	97.09	RGP-3	91.56	Acc. 1639	89.43	W-302	87.25	W-172	70.84
Acc. 1660	92.6	W-085	91. 22	Acc. 1644	85.72	W-396	86.33	W-208	63.49
Acc. 1667	92.72	W-306	93.49	Acc. 1651	89.79	W-408	79.37	W-355	52.3
Acc. 1696	94.71	W-344	96.68	Acc. 1653	78.98	W-453	80.38	W-395	72.07
Acc. 1702	96	W-361	94.38	Acc. 1658	80.18	W-504	89.77		
Acc. 1709	94.06	W-448	94.48	Acc. 1663	79.49	W-507	82.1		
				Acc. 1664	76.95	W-517	90.69		

#### Principal Component Analysis for Waterlogging Tolerance Response

**FIGURE 1:**
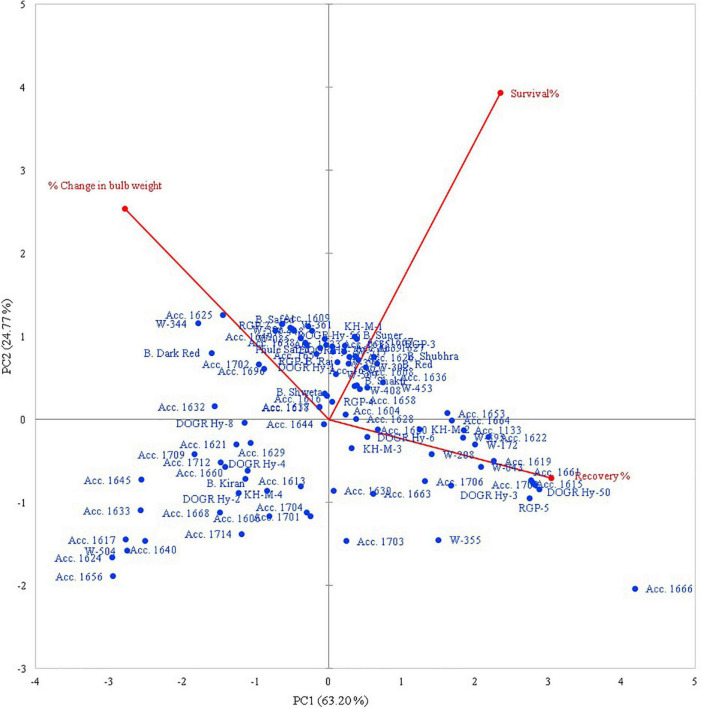
Principal component of the genotypic trait grouping under waterlogging condition.

**FIGURE 2:**
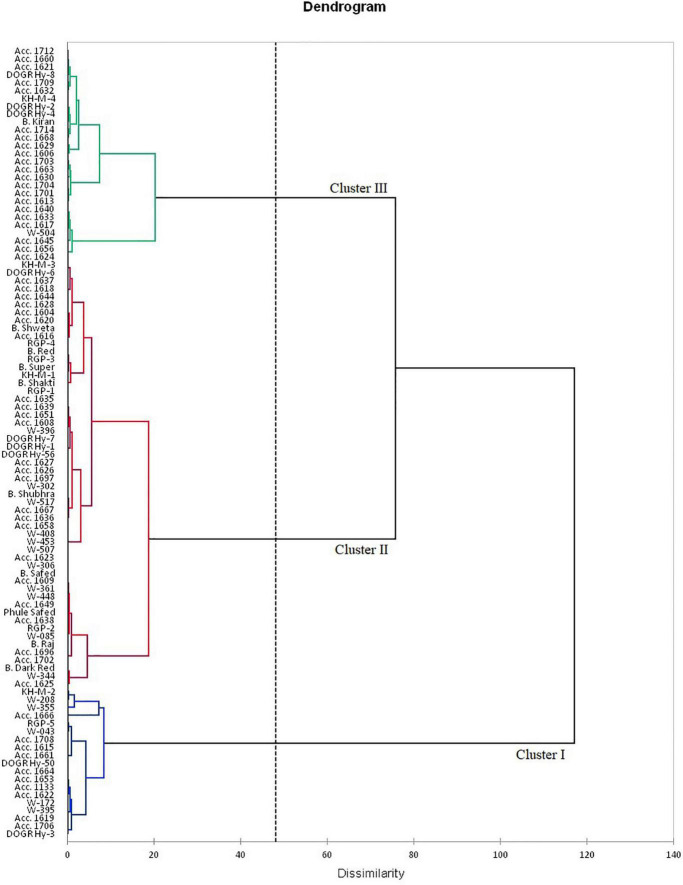
Dendrogram using Ward’s method for distributing 100 onion genotypes.

The rotated component matrix demonstrated the variability exhibited by different PCs and their association with the evaluated parameters. The principal component analysis (PCA) results indicate that the PC 1 group had an Eigenvalue of >1 (1.89), contributing to 63.2% variability. The association between the studied traits and the 100 genotypes with different PC groups (PC 1 and PC 2) is illustrated using PC biplots for the waterlogging condition (Figure 1). The angle between the dimension vectors represents the association among the plant survival rate, recovery percentage, and percentage change in bulb weight, which was used for categorizing the genotypes into highly tolerant, moderately sensitive, and highly sensitive groups. Genotypes advanced in a specific trait were plotted in the direction along or near the vector line. Under waterlogging stress, most of the genotypes were distributed on the positive side of the PC 1 and PC 2 groups. Genotypes such as Acc. 1666, RGP-5, DOGR Hybrid 50, and W-355 were more inclined toward the recovery percentage direction, and these genotypes exhibited 100% plant survival and recovery with 36.2–61.4% bulb weight reduction under waterlogged conditions. Hence, these genotypes were classified as waterlogging-tolerant genotypes. All the highly tolerant genotypes (19 genotypes) with high survival and recovery percentage under waterlogging stress were grouped in cluster I (Figure 2 and Supplementary Table 2). Furthermore, Acc. 1622, W-208, RGP-5, and KH-M-2 genotypes exhibited 100% survival, 90.0–96.7% recovery, and 57.3–74.1% bulb weight reduction under waterlogging stress and, hence, were characterized as tolerant genotypes. Twenty-seven genotypes classified as tolerant were grouped in cluster III. By contrast, the W-344, Acc. 1625, Bhima Dark Red, and Acc. 1702 genotypes were distributed more on the positive side of the percentage change in bulb weight and, thus, were classified as highly sensitive genotypes. These genotypes also exhibited poor recovery after relieving waterlogging stress. Similarly, Acc. 1656, Acc. 1624, W-504, Acc. 1617, and Acc. 1640 genotypes demonstrated inclination toward the negative side of both PC 1 and PC 2 with a poor plant survival rate. All these genotypes were classified as highly sensitive genotypes (54 genotypes) for waterlogging and grouped in cluster II (Figure 2). Furthermore, all the onion cultivars, namely, Bhima Super, Bhima Raj, Bhima Red, Bhima Shakti, Bhima Dark Red, Bhima Shweta, Phule Safed, Bhima Safed, and Bhima Shubhra except Bhima Kiran, were located in Cluster II. This result confirmed the sensitivity of popular onion varieties to waterlogging stress. Highly sensitive genotypes W-344 and Acc. 1702 of Cluster II exhibited high plant mortality and poor bulb development under waterlogging conditions. Based on the relative performance of the studied genotypes under waterlogging condition, 12 contrasting genotypes (six tolerant genotypes, namely, Acc. 1666, Acc. 1622, W-355, W-208, KH-M-2, and RGP-5; five sensitive genotypes, namely, W-344, W-361, W-085, W-448, and Acc. 1639; and an intermediate genotype Acc. 1630) were selected to identify onion genotypes with promising waterlogging-tolerant traits.

### Experiment 2. Response of Identified Contrasting Genotypes to Waterlogging Stress

#### Analysis of Variance

Three-way analysis of variance showed that PH was significantly affected by genotypes, waterlogging stress, year, and year × genotype interaction (Table 4). However, number of leaves, pyruvic acid, TSS, phenol, antioxidant activity, chlorophyll, MSI, and root length/shoot length ratio were significantly affected by year, waterlogging, genotypes, and their interaction. Furthermore, leaf area and onion yield were significantly affected by year, treatment, genotypes, year × treatment, year × genotype, and genotype × treatment. However, their three-way interaction was not significant.

**TABLE 4:** Combined ANOVA (mean square) for morphological, physiological, biochemical, and yield traits under well-watered and waterlogging conditions.

Source of variation	DF	PH	NL	LL	LA	CHL	MSI	AOX	PHE	PY	TSS	R:S ratio	YLD
Year	1	172.18*	59.20**	14.87	446.68*	18.23**	0.037	3.04**	86.86**	1.04*	5.22**	0.053**	89.68*
Replication	2	1.081	0.366	3.261	5.047	0.0038	5.860	0.141	0.582	0.029	0.022	0.0007*	2.510
Environment	1	948.31**	246.31**	926.23**	1107.85**	44.73**	7117.10**	5.576**	14.66**	5.803**	114.09**	0.042**	5436.61**
Genotypes	11	127.11**	4.376**	40.08**	56.67**	3.747**	119.02**	1.104**	2.720**	0.663**	2.234**	0.034**	97.69**
Year × Environment	1	14.06	2.87*	20.71	1.59	0.241*	65.35**	0.014	0.864	0.166*	5.28**	0.012*	28.19*
Year × genotypes	11	62.77**	2.292**	72.33**	22.91**	2.292**	39.26**	1.276**	5.675*	1.515**	0.999**	0.004**	21.70**
Environment × genotypes	11	12.08	3.746**	38.24**	18.09**	0.708**	71.40**	0.185**	0.309	0.379**	1.085**	0.020**	37.18**
Year × Environment × genotypes	11	5.83	1.09**	37.73**	3.65	0.37**	18.97**	0.48**	0.39*	0.43**	1.28**	0.009**	4.97
Error	88												

* Indicates significance at the 0.05 level of significance.

** Indicates significance at the 0.01 level of significance.

*PH, plant height; NL, number of leaves per plant; LL, leaf length; LA, leaf Area; PHE, total phenol; AOX, antioxidant activity; PY, pyruvic acid; TSS, total soluble solids; CHL, total chlorophyll; MSI, membrane stability index; YLD, bulb yield; R:S, root to shoot ratio, DF, degree of freedom.*

#### Morpho-Physiological and Biochemical Response of Contrasting Genotypes Under Waterlogging Stress

Plant height of the genotypes decreased significantly (5–22%) under waterlogged conditions compared with well-watered conditions (control). Genotypes W-355 and KH-M-2 exhibited significantly higher PH than other genotypes under waterlogging stress (Table 5). PH of all genotypes in both the waterlogged condition and control were higher in 2018 compared with the value recorded in 2019. Phenotypic traits of all the genotypes, such as leaf number, LL, and LA decreased under waterlogging conditions compared with those of the control in both years. Under waterlogged conditions, the maximum number of leaves was recorded in Acc. 1666 in both years, whereas W-344 and W-361 exhibited higher reduction in these traits than other genotypes. Genotypes Acc.1666, KH-M-2, W-355, W-208, and RGP-5 had significantly less LA reduction under stress conditions both in 2018 and 2019 compared with other genotypes. Minimum LA under stress was recorded in genotypes W-085 and W-344 in both years. The root–shoot ratio was higher in W-208, RGP-5, and KH-M-2, whereas it was lowest in Acc. 1639 under stress conditions in both years. Genotypes, namely, Acc. 1666, Acc. 1622, RGP-5, W-208, and KH-M-2, were statistically at par for leaf number, LL, LA, and root–shoot ratio under stress conditions in both years. Furthermore, waterlogging stress significantly affected the total phenol, antioxidant activity, pyruvic acid, TSS, chlorophyll, and MSI of the genotypes in both years (Table 4). Waterlogging stress decreased TSS by 6.5–21.8%, chlorophyll content by 3.2–46.8%, and MSI by 6.2–33.6% compared with the control treatment (Table 7). The highest chlorophyll content reduction was recorded in W-344 (46.8% in 2018 and 37.2% in 2019), and the lowest reduction was observed in Acc. 1666 (8.5% in 2018 and 3.2% in 2019) under stress conditions compared with the control treatment. Furthermore, Acc. 1622 exhibited a significantly higher MSI with minimum chlorophyll content reduction under stress conditions in both years. All the genotypes under stress conditions exhibited lower TSS and higher phenol, pyruvic acid concentrations, and antioxidant activity in comparison to the control treatment (Table 6). Furthermore, percentage increase in total phenol and pyruvic acid contents in sensitive genotypes W-344, W-361, W-448, Acc. 1639, and W-085 under stress conditions were slightly higher than the control plants. Overall, TSS, total phenol, pyruvic acid, and antioxidant activity were significantly lower in all genotypes in 2018 under both stress and control conditions than that of the values recorded in 2019.

**TABLE 5:** Phenotypic and yield traits of contrasting onion genotypes evaluated under well-watered and waterlogging conditions.

Genotype	Plant height				Number of leaves				Leaf length				Leaf area				Root/shoot length ratio			
	2018		2019		2018		2019		2018		2019		2018		2019		2018		2019	
	WW	WL	WW	WL	WW	WL	WW	WL	WW	WL	WW	WL	WW	WL	WW	WL	WW	WL	WW	WL
Acc. 1622	54.50	50.94	48.67	45.71	7.11	3.44	8.33	7.33	32.13	26.93	37.46	32.19	27.73	23.26	28.03	24.57	0.69	0.66	0.61	0.73
Acc. 1630	51.54	48.53	51.04	43.67	7.44	5.33	7.33	5.33	32.47	31.07	33.71	29.71	24.21	21.21	29.36	21.17	0.71	0.49	0.59	0.49
Acc. 1639	55.07	51.78	51.52	45.41	6.89	3.67	8.33	4.67	35.50	35.10	39.27	29.81	25.79	15.63	36.05	25.58	0.58	0.46	0.56	0.49
Acc. 1666	53.17	50.51	54.23	49.42	7.22	6.33	8.67	7.67	32.83	42.40	38.88	33.17	26.11	20.74	30.04	26.14	0.70	0.60	0.54	0.68
KH-M-2	56.90	53.58	50.64	46.53	7.22	5.11	7.67	5.33	30.03	32.10	36.93	30.74	24.50	22.03	24.24	22.60	0.64	0.73	0.63	0.69
RGP-5	54.04	48.93	47.47	45.29	6.67	5.56	7.33	5.33	38.23	31.90	37.94	32.74	21.85	19.72	29.32	25.63	0.69	0.74	0.68	0.68
W-085	53.77	49.14	52.67	40.99	8.11	3.00	8.33	5.67	43.07	35.03	34.62	28.83	20.77	14.39	25.86	17.52	0.65	0.56	0.60	0.57
W-208	37.63	33.64	46.83	43.69	6.78	4.67	9.11	7.67	27.43	26.17	36.72	33.59	23.56	21.93	27.68	22.65	0.67	0.76	0.63	0.66
W-344	50.47	42.01	52.44	44.47	6.56	3.78	8.11	5.33	37.70	26.67	38.22	29.83	22.49	14.80	24.24	17.70	0.66	0.68	0.59	0.48
W-355	57.43	54.13	51.60	48.79	8.22	5.56	9.33	8.33	39.37	36.90	35.06	29.46	25.56	20.47	29.12	25.40	0.62	0.66	0.66	0.61
W-361	51.31	46.43	51.12	42.80	7.89	3.11	8.67	5.33	40.93	29.80	36.29	28.97	24.83	17.43	26.98	21.51	0.69	0.51	0.64	0.53
W-448	51.94	44.04	50.79	43.18	8.56	4.33	9.44	4.67	47.57	31.43	33.56	29.64	25.28	17.04	26.56	17.93	0.70	0.51	0.58	0.51

*WW, well-watered; WL, waterlogging stress; LSD, least significant difference.*

*LSD and p-value are presented in Supplementary Table 3.*

**TABLE 6:** Biochemical traits of contrasting onion genotypes evaluated under well-watered and waterlogging conditions.

Genotype	TSS				Phenol				Pyruvic acid				AOX			
	2018		2019		2018		2019		2018		2019		2018		2019	
	WW	WL	WW	WL	WW	WL	WW	WL	WW	WL	WW	WL	WW	WL	WW	WL
Acc. 1622	13.73	12.80	15.29	11.81	4.17	5.05	6.68	7.40	2.78	2.65	1.87	2.02	1.65	2.14	1.39	2.25
Acc. 1630	14.40	13.27	15.27	12.68	4.97	6.02	7.63	7.22	1.37	2.33	2.41	2.26	2.25	2.32	1.35	1.7
Acc. 1639	14.57	12.80	15.04	11.90	6.18	6.37	6.40	7.10	1.83	2.78	2.69	2.75	2.14	2.62	2.61	2.71
Acc. 1666	14.30	12.97	13.08	12.57	5.10	5.12	4.97	5.45	1.69	2.29	2.98	3.22	2.4	1.91	1.8	2.25
KH-M-2	14.60	13.43	14.84	12.68	3.95	4.72	6.65	6.70	2.61	2.89	1.2	2.87	1.93	2.35	1.29	1.78
RGP-5	14.83	12.97	15.11	12.58	5.15	6.18	5.22	6.03	2.03	2.33	2.92	3.04	1.99	2.08	2.6	3.78
W-085	15.67	14.10	15.01	12.47	3.33	5.48	7.22	7.92	1.87	2.29	3.77	3.09	1.87	2.26	2.42	2.88
W-208	15.70	13.27	15.95	13.28	5.80	6.35	5.50	6.02	1.73	2.37	2.14	2.53	2.21	2.52	1.99	2.53
W-344	13.77	13.07	13.51	12.33	5.45	6.37	7.33	7.65	1.8	2.41	1.66	2.89	2.12	2.79	2.76	2.96
W-355	14.80	13.73	13.41	12.76	4.80	5.47	5.07	6.05	2.14	2.27	1.67	2.01	1.29	2.27	2.92	1.85
W-361	13.97	13.40	15.79	12.1	3.35	4.40	7.37	7.52	2.97	2.77	2.08	2.29	1.56	2.08	2.68	3.62
W-448	16.03	13.80	14.11	13.29	4.12	4.37	6.83	7.62	1.56	2.63	1.87	2.3	1.85	2.4	2.71	3.18

*AOX, antioxidant activity; TSS, total soluble solids; WW, well-watered; WL, waterlogging stress; LSD, least significant difference.*

*LSD and p-value are presented in Supplementary Table 3.*

**TABLE 7:** Physiological traits of contrasting onion genotypes evaluated under well-watered and waterlogging conditions.

Genotype	MSI				Chlorophyll				Yield (t ha^–1^)			
	2018		2019		2018		2019		2018		2019	
	WW	WL	WW	WL	WW	WL	WW	WL	WW	WL	WW	WL
Acc.1622	71.69	65.82	73.20	68.68	4.38	4.15	6.70	5.18	20.78	9.91	18.36	8.87
Acc.1630	70.70	56.54	68.05	49.84	5.04	4.06	4.06	2.70	24.87	8.76	13.48	4.75
Acc. 1639	68.50	52.10	67.15	50.88	4.57	3.56	5.08	3.22	23.56	5.21	17.92	4.67
Acc. 1666	74.23	57.70	71.29	63.34	4.46	4.08	4.44	4.30	26.04	16.41	24.19	15.52
KH-M-2	63.04	56.69	68.51	60.16	4.61	3.63	5.82	5.36	23.13	11.05	17.78	8.55
RGP-5	65.86	54.15	71.44	58.55	4.16	3.60	6.29	5.21	18.03	8.54	17.63	7.71
W-085	70.45	59.23	70.45	49.79	4.21	2.56	5.38	4.32	19.32	4.24	20.21	5.72
W-208	66.44	57.72	70.56	58.92	4.96	4.02	5.84	4.71	14.23	8.73	14.88	9.29
W-344	67.34	59.07	67.49	49.19	4.47	2.38	4.46	2.80	13.18	3.67	17.01	5.11
W-355	73.52	54.80	77.18	57.31	4.35	3.33	5.15	4.78	27.48	11.38	23.72	9.04
W-361	70.59	52.97	71.05	48.33	4.61	3.33	5.98	3.42	22.81	5.32	21.27	5.50
W-448	68.20	51.21	69.98	46.48	3.80	2.52	3.94	2.78	22.62	4.74	20.05	4.93

*WW, well-watered; WL, waterlogging stress.*

*LSD and p-value are presented in Supplementary Table 3.*

#### Yield Response of the Selected Genotypes Under Waterlogging Stress

Total bulb yield was significantly reduced (35–78%) in all the tested genotypes under stress conditions compared with that of the control (Table 7). Of all the genotypes, Acc. 1666 exhibited a comparatively lower reduction in bulb yield under stress conditions and demonstrated high bulb yield under both stress and well-watered conditions in both years. Similarly, W-355 produced bulb yield at par with Acc. 1666 under stress and control conditions. Bulb yield reduction in sensitive genotypes (W-344, W-085, W-361, W-448, Acc. 1639) under waterlogged conditions was >70% compared with the control condition. The lowest bulb yield was observed in W-344. Genotype Acc. 1630 exhibited a relatively higher yield than sensitive genotypes under stress.

#### Association Among Different Traits Under Waterlogged and Well-Watered Conditions

Furthermore, the bulb yield and MSI were positively correlated with the number of leaves, LL, LA, and chlorophyll concentration and negatively correlated with total phenol, pyruvic acid, and antioxidant activity (Table 8). A positive correlation was observed between bulb yield and MSI. Leaf area was positively correlated with PH, leaf length, number of leaves, total chlorophyll, and MSI. Vital stress-tolerant traits, root–shoot ratio were positively correlated with the bulb yield, total chlorophyll, and MSI of plants.

**TABLE 8:** Pearson correlation coefficient among the studied traits in contrasting genotypes under control and waterlogging conditions.

Parameters	PH	NL	LL	LA	PHE	AOX	PYR	TSS	CHL	MSI	YLD	RL/SL
**PH**	1.000	0.362**	0.469**	0.337**	−0.376**	−0.320**	−0.105	0.302*	0.168	0.490**	0.595**	0.001
**NL**		1.000	0.482**	0.686**	−0.091	−0.253	−0.289*	0.492**	0.608**	0.710**	0.737**	0.24
**LL**			1.000	0.297*	−0.332**	−0.291*	−0.204	0.477**	0.265	0.446**	0.521**	0.12
**LA**				1.000	−0.043	−0.172	−0.073	0.354**	0.629**	0.651**	0.608**	0.188
**PHE**					1.000	0.329**	0.044	−0.294*	0.0042	−0.317*	−0.404**	−0.309*
**AOX**						1.000	0.182	−0.340**	−0.194	−0.373**	−0.350**	−0.187
**PYR**							1.000	−0.343**	−0.124	−0.325**	−0.291**	−0.17
**TSS**								1.000	0.416**	0.606**	0.585**	0.193
**CHL**									1.000	0.652**	0.521**	0.369**
**MSI**										1.000	0.826**	0.428**
**YLD**											1.000	0.325**
**SLRL**												1.000

* Indicates significance at the 0.05 level of significance.

** Indicates significance at the 0.01 level of significance.

*PH, plant height; NL, number of leaves per plant; LL, leaf length; LA, leaf Area; PHE, total phenol; AOX, antioxidant activity; PY, pyruvic acid; TSS, total soluble solids; CHL, total chlorophyll; MSI, membrane stability index; YLD, bulb yield; RL/SL, root to shoot ratio.*

#### Descriptive Statistics

Based on the Euclidean distance among the traits, we classified 12 genotypes into two clusters using Ward’s method (Table 9). The mean value of waterlogging-tolerant attributes, namely, PH, number of leaves, LL, LA, chlorophyll content, MSI, and bulb yield, was higher in Cluster I than in Cluster II. Genotypes of Cluster I exhibited the highest mean values for MSI (58.59), followed by PH (47.38 cm). The mean value of bulb yield in Cluster I genotypes (9.89) was approximately 50% higher than that in Cluster II (4.91). Divergence analysis revealed that, among the various parameters, bulb yield accounted for the maximum (14.91%) genetic diversity, followed by LA (13.00%) (Table 10). Among the biochemical traits, the total chlorophyll content exhibited the maximum genetic diversity (12.34%). The genotypic coefficient of variation (GCV), phenotypic coefficient of variation (PCV), heritability, and genetic advancement are presented as the percentage of the mean of physiological, biochemical, and yield traits (Table 11). The value of PCV was more than that of GCV for all the studied parameters, which reflected the influence of environmental factors on the expression of these traits. Bulb yield exhibited the maximum estimated values for the GCV (42.56) and PCV (42.90), followed by total chlorophyll content (18.86 and 18.92 of GCV and PCV, respectively). The TSS exhibited the minimum estimated values for GCV and PCV of 2.70 and 2.95, respectively. Heritability values did not vary significantly among the studied traits, and the lowest value was exhibited by TSS (0.84) and total phenols (0.85). The MSI demonstrated the maximum (5%) value for genetic advancement of 9.93, whereas the percentage increase over the mean (5%) was highest for bulb yield (86.99).

**TABLE 9:** Descriptive statistics for various parameters of 12 contrasting genotypes under different clusters.

Parameters	Cluster I		Cluster II	
	Mean	SD	Mean	SD
**PH**	47.38	04.26	45.03	02.13
**NL**	05.92	00.78	04.35	00.17
**LL**	32.08	02.84	30.51	01.74
**LA**	22.68	00.88	17.95	02.03
**PHE**	05.98	00.43	06.47	00.47
**AOX**	02.26	00.34	02.75	00.13
**PYR**	02.50	00.27	02.61	00.12
**TSS**	12.91	00.33	12.93	00.48
**CHL**	04.22	00.42	03.09	00.43
**MSI**	58.59	04.46	51.93	02.39
**YLD**	09.89	02.91	04.91	00.37
**R:S ratio**	00.65	00.08	00.53	00.04

*PH, plant height; NL, number of leaves per plant; LL, leaf length; LA, leaf Area; PHE, total phenol; AOX, antioxidant activity; PY, pyruvic acid; TSS, total soluble solids; CHL, total chlorophyll; MSI, membrane stability index; YLD, bulb yield; R:S, root to shoot ratio.*

**TABLE 10:** Contribution of various parameters toward genetic divergence in 12 contrasting onion genotypes.

Parameters	Contribution (%)
**PH**	05.45
**NL**	12.17
**LL**	06.43
**LA**	13.00
**PHE**	09.06
**AOX**	08.42
**PY**	00.10
**TSS**	00.26
**CHL**	12.34
**MSI**	09.71
**YLD**	14.91
**R:S ratio**	08.08

*PH, plant height; NL, number of leaves per plant; LL, leaf length; LA, leaf Area; PHE, total phenol; AOX, antioxidant activity; PY, pyruvic acid; TSS, total soluble solids; CHL, total chlorophyll; MSI, membrane stability index; YLD, bulb yield; R:S, root to shoot ratio.*

**TABLE 11:** Estimate of genetic parameters for 12 quantitative traits of 12 contrasting onion genotypes.

Parameters	GCV	PCV	h2 (Broad sense)	Genetic advancement 5%	Genetic advancement as % of mean 5%
**PH**	07.47	07.78	0.92	6.86	14.79
**NL**	17.91	18.91	0.90	1.84	34.94
**LL**	07.03	07.89	0.79	4.06	12.91
**LA**	13.30	13.53	0.97	5.58	26.96
**PHE**	07.43	08.06	0.85	0.87	14.09
**AOX**	14.01	14.72	0.91	0.68	27.48
**PY**	08.28	08.71	0.90	0.41	16.22
**TSS**	02.70	02.95	0.84	0.66	05.08
**CHL**	18.86	18.92	0.99	1.45	38.71
**MSI**	08.77	08.90	0.97	9.93	17.79
**YLD**	**42.56**	**42.90**	0.98	6.80	86.99
**R:S ratio**	14.93	15.15	0.97	0.18	30.32

*PH, plant height; NL, number of leaves per plant; LL, leaf length; LA, leaf Area; PHE, total phenol; AOX, antioxidant activity; PY, pyruvic acid; TSS, total soluble solids; CHL, total chlorophyll; MSI, membrane stability index; YLD, bulb yield; R:S, root to shoot ratio, GCV, genetic coefficient of variance; PCV, phenotypic coefficient of variance; h^2^, heritability. Bold indicating maximum variability.*

#### Principal Component Biplot Analysis

Principal component analysis revealed the relative contribution of various parameters (morphological, physiological, biochemical, and yield traits) for evaluating the genetic diversity among the 12 contrasting onion genotypes under waterlogging conditions. The PCA analysis produced three PC groups with an eigenvalue of more than 1, accounting for 76.63% variability (PC 1, 50.19%; PC 2, 13.59%; and PC 3, 12.95%) (Table 12). All the studied parameters contributed positively to the PC groups. In the PC 1 group, bulb yield (0.898) accounted for the highest variability, followed by LA (0.784), total chlorophyll content (0.744), and the number of leaves per plant (0.733). Conversely, pyruvic acid (0.006) and TSS (0.016) exhibited the least variability in the PC 1 group. The maximum variability of TSS (0.518) and pyruvic acid (0.497) was recorded in the PC 2 and PC 3 groups, respectively. Overall, the PC 1 group exhibited the maximum variability for waterlogging tolerance–associated parameters compared with the PC 2 and PC 3 groups.

**TABLE 12:** Rotated component matrix for principal components of 12 traits of 12 contrasting onion genotypes evaluated under waterlogging condition.

Parameters	PC-1	PC-2	PC-3
**PH**	0.329	0.013	0.318
**NL**	0.733	0.080	0.064
**LL**	0.388	0.195	0.287
**LA**	0.784	0.029	0.004
**PHE**	0.546	0.154	0.004
**AOX**	0.508	0.052	0.004
**PY**	0.006	0.056	**0.497**
**TSS**	0.016	**0.518**	0.222
**CHL**	0.744	0.118	0.014
**MSI**	0.585	0.259	0.028
**YLD**	**0.898**	0.020	0.006
**R: S ratio**	0.487	0.139	0.105
**Eigenvalue**	06.024	01.631	01.555
**Variability (%)**	50.197	13.592	12.955
**Cumulative variance (%)**	50.197	63.790	76.745

*PH, plant height; NL, number of leaves per plant; LL, leaf length; LA, leaf Area; PHE, total phenol; AOX, antioxidant activity; PY, pyruvic acid; TSS, total soluble solids; CHL, total chlorophyll; MSI, membrane stability index; YLD, bulb yield; R:S, root to shoot ratio. Bold indicating maximum variability.*

**FIGURE 3:**
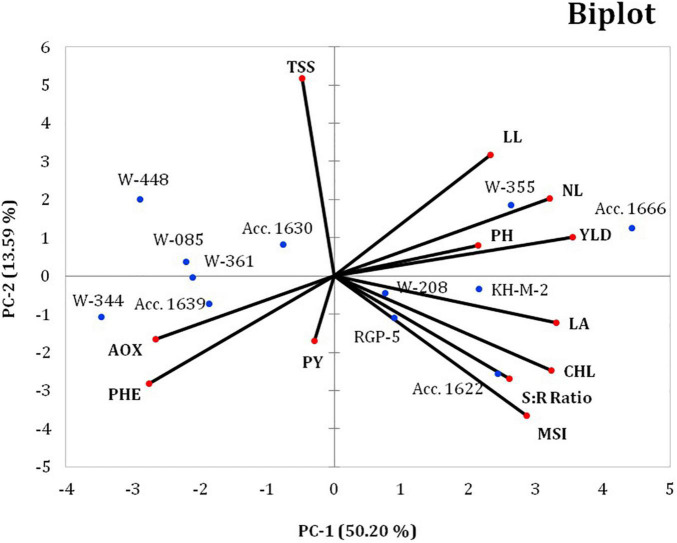
Principal component (PC) iplot for the 12 different traits of 12 contrasting onion genotypes under waterlogging conditions. PH, plant height; NL, number of leaves per plant; LL, leaf length; LA, leaf area; PHE, total phenol; AOX, antioxidant activity; PY, pyruvic acid; TSS, total soluble solids; CHL, total chlorophyll; MSI, membrane stability index; YLD, bulb yield; R:S, root-to-shoot ratio.

Furthermore, the biplot was plotted using a two -way matrix (genotype × parameter) of 12 different traits and 12 contrasting genotypes under waterlogging conditions, which indicates the relationship among different traits (physiological, biochemical, and yield) and the genotypes with particular PCs (Figure 3). The first two axes of the biplot explain the variability of 50.20 and 13.59% by PC 1 and PC 2, respectively. The genotypes were toward the positive and negative sides of the PC 1 and PC 2 groups across the axis. The genotypes, namely, Acc. 1666 and W-355, had PC scores >0 and were located on the positive side of PC 1 and PC 2; these genotypes were inclined toward the direction of useful traits, such as bulb yield, PH, number of leaves, and LL. Similarly, genotypes KH-M-2, W-208, RGP-5, and Acc. 1622 were located on the positive side of the PC 1 group and were inclined toward the direction of traits such as LA, chlorophyll content, membrane stability, and root–shoot ratio and were, therefore, classified as waterlogging-tolerant genotypes with promising stress adaptive traits. Genotypes W-344, W-361, and Acc. 1639 were inclined away from waterlogging-tolerant traits on the negative side of PC 1 and were, thus, designated as waterlogging-sensitive genotypes. Overall, PCA biplots helped in confirming the waterlogging-tolerant onion genotypes using traits bulb yield, PH, number of leaves, LL, LA, chlorophyll, MSI, and root–shoot ratio.

## Discussion

Cultivation of monsoon season onion in India coincides with the southwest monsoon season (June–October), during which approximately 98% of rainfall is received. Excess and uneven distribution of rain during the monsoon season causes waterlogging. Waterlogging adversely affects the performance of many crops. The onion crop is highly sensitive to waterlogging due to its shallow root system. This sensitivity varies with the plant developmental stage and genotype (Dubey et al., 2020). Our previous studies reveal that the bulb development phase is the most sensitive growth period for waterlogging stress in onion crops (Ghodke et al., 2018). Furthermore, yield loss in onion crop due to waterlogging varies from 50 to 70% in monsoon onion (Samra et al., 2006). Hence, the identification of stress-tolerant genotypes is imperative to overcome the unfavorable effects of waterlogging stress. In the present study, 100 onion genotypes were screened, and 12 contrast onion genotypes were evaluated under well-watered and waterlogged conditions to identify waterlogging-tolerance traits in onion.

### Experiment 1

In the present study, traits such as plant survival, recovery, and bulb weight were considered for preliminary screening. In other studies, plant survival (Wu et al., 2017), recovery, and plant biomass (Ploschuk et al., 2017) are considered for screening waterlogging-tolerant genotypes in different crops. The recovery after stress involves the distribution of the carbon to roots after waterlogging stress and hypoxia conditions to enhance root growth and to reestablish a root-to-shoot ratio during the recovery phase (Cotrozzi et al., 2021). High plant mortality among sensitive genotypes under waterlogged conditions may be attributed to anoxic conditions created by waterlogging. Anoxic conditions probably damage the root tissues, thereby inhibiting aerobic respiration, energy generation, and nutrient acquisition (Pan et al., 2021). Ploschuk et al. (2017) reports the death of root tissues in *Bromus catharticus* due to waterlogged conditions. The hypoxia–induced nutrient deficiency or toxicity may have affected plant physiological processes, such as photosynthesis, respiration, and plant growth, thereby causing plant death (Najeeb et al., 2015). However, in the current study, 54 genotypes exhibited 100% plant survival under waterlogged conditions for 10 days, which could be due to the formation of aerenchymatic root cells. Development of aerenchyma cells along the root axes under waterlogged conditions, particularly in grasses, facilitates oxygen diffusion (Jackson and Drew, 1984), which, in turn, facilitates respiration and water and nutrient uptake (Ploschuk et al., 2018).

Furthermore, the detrimental effect of waterlogging was evident among sensitive genotypes during recovery in the present study. Of the 54 genotypes, 43 genotypes exhibited higher plant mortality during recovery, which could be due to the combined effect of reduced root growth and death of root tissues during waterlogging (Ploschuk et al., 2017). The adaptive mechanisms exhibited by these genotypes were insufficient to overcome the stress, and hence, these genotypes were classified as waterlogging-sensitive genotypes. Sensitive genotypes could not be used for cultivation during the monsoon season. Furthermore, 16 genotypes successfully recovered from stress with more than 90% survival and showed lesser reduction in plant and root growth in the present study. The positive relationship observed between plant height, number of leaves, leaf length, leaf area, and bulb yield in the present study indicates that the reduction in plant and root growth significantly affects bulb size and weight. Pampana et al. (2016) also reports that waterlogging for 5 days decreased root dry weight by 35% and rootlets by 25% compared with the control plants in pea and white lupin. Similar reductions in root and plant biomass of waterlogged plants were observed by Ploschuk et al. (2017) and Masoni et al. (2016). In our previous study, the waterlogging condition during bulb initiation and development significantly reduced plant growth, bulb size, and onion yield (Ghodke et al., 2018). In the present study, a phenotypic approach for genotype screening was further supported by PCA, through which 16 genotypes exhibited 100% plant survival and more than 90% recovery with better bulb size and were classified as waterlogging-tolerant genotypes. Sundgren et al. (2018) also used PCA as a potential statistical method for ranking the wheat genotypes based on their tolerance level under waterlogging conditions.

### Experiment 2 Effect of Waterlogging Stress on Physiological and Biochemical Traits

Twelve contrasting onion genotypes were evaluated to understand the phenotypic, physiological, and biochemical changes under the waterlogging condition in 2018 and 2019. These genotypes displayed a reduction in the PH, number of leaves, LA, and LL, which could be due to waterlogging-induced anoxic conditions. The reduced photosynthetic CO_2_ assimilation rate and water and nutrient uptake by the damaged roots under waterlogged conditions might have contributed to the reduced plant growth and development (Arduini et al., 2019). Similar reductions in LA, LL, and leaf number in waterlogging-tolerant genotypes are reported in soybean (Henshaw et al., 2007) and mung bean (Ahmed et al., 2002; Kumar et al., 2013) as a mechanism to avoid water loss and adapt to the waterlogging environment. The strong association of LA with total chlorophyll and leaf number in the present study revealed that maintaining the vegetative growth is one of the survival mechanisms used by the stress-tolerant genotypes to adapt to adverse environment. This observation confirmed that plants could withstand the waterlogged condition by maintaining their photosynthetic activity and aerial vegetative growth. The genotypes possessing these traits had a better waterlogging-tolerance than the other genotypes. The sensitive genotypes (W-344, W-361, W-085, W-448, and Acc. 1639) failed to produce new leaves and exhibited high leaf senescence, leading to plant mortality. Anoxic conditions in the rhizosphere restricts oxygen uptake in plants (Debnath et al., 2021; Pan et al., 2021). Pan et al. (2021) also reports that the plants could maintain energy production through glycolysis and ethanol fermentation under waterlogged conditions; however, prolonged waterlogging leads to the accumulation of toxic substances and reactive oxygen species, which causes cell death and plant senescence. The pigment degradation affects the photosynthetic activity results in the slow-down of the crop growth, which directly affects the yield (Cotrozzi et al., 2021). Inhibition of plant growth and development is also reported in sensitive genotypes of field bean (Pociecha et al., 2008), tomato (De Ollas et al., 2021), and mung bean (Ahmed et al., 2002; Kumar et al., 2013). Root decay in sensitive genotypes caused by waterlogging could be the possible reason for poor tolerance to waterlogging (Palta et al., 2010). However, waterlogging-tolerant genotypes exhibited a higher number of leaves, LA, LL, and shoot–root ratio than sensitive genotypes, which could be due to the ability of the stress-tolerant plants to withstand waterlogged conditions by producing aerenchyma cells. Similarly, a low degree of root decay and aerenchyma formation has been reported in cow pea (Hong et al., 1977) and faba bean (Al-Amri, 2019) as a mechanism to acquire stress tolerance under waterlogging conditions. Aerenchyma cells in stress-tolerant genotypes facilitate oxygen transport from root to shoot (Colmer, 2002).

The MSI is used as stress-tolerance indicator in plants (Kumar et al., 2013). Higher membrane stability recorded in stress-tolerant genotypes compared with that in sensitive genotypes in the present study indicates the existence of a stress-tolerance mechanism. Premachandra et al. (1992) also report a correlation between high MSI and plant stress tolerance. Jackson et al. (1982) report membrane damage due to oxygen deficiency and solute leakage up to 40 times in peas under stress conditions. Furthermore, damaged root tips and decreased root growth under waterlogged conditions stimulate leaf chlorosis and inhibit the aerial growth of plants (Drew, 1983), which is evident from the low total chlorophyll concentration recorded in the present study. A similar reduction in chlorophyll concentration under waterlogged conditions is reported in onion (Ghodke et al., 2018), mung bean (Kumar et al., 2013), and cucumber (Barickman et al., 2019). A strong association of RL/SL with the total chlorophyll concentration and membrane stability implies the vital role of physiological traits of plants in waterlogging tolerance. Furthermore, the increase in total phenol and antioxidant activity under waterlogged conditions may enable the plants to adapt to the waterlogged condition. Andrade et al. (2018) report that waterlogging-resistant soybean genotypes exhibit tolerance by increasing their antioxidant and net photosynthetic activities and reducing the reactive oxygen species production and cell membrane damage. Similarly, an increase in total phenol and antioxidant activity is reported against drought (Ghodke et al., 2020), salt (Jahangir et al., 2020), and temperature stress (Shamloo et al., 2017; Thangasamy et al., 2021). Additionally, in the current study, the higher total phenol and antioxidant activity observed in sensitive genotypes compared with those in tolerant genotypes indicate that the total phenol production and antioxidant activity in sensitive genotypes might increase in response to waterlogged conditions. The stress response from the plants may induce a partial stress tolerance, which potentially allows the recovery after waterlogging stress (Barickman et al., 2019). However, the adaptive mechanism exhibited by the sensitive genotypes may be insufficient to overcome the effect of stress; hence, these genotypes were classified as waterlogging-sensitive genotypes. Consistent with these findings, the total phenol concentration under waterlogged conditions in maize was reported to be high by Jaiswal and Srivastava (2018). Furthermore, higher TSS, phenol, pyruvic acid, and antioxidant activity observed in 2019 compared to 2018 could be attributed to the exposure of onion genotypes to artificial waterlogging and excess rainfall during the growing period.

### Effect of Waterlogging Stress on Bulb Yield

Bulb yield in onion crops is a valuable trait for selecting genotypes under waterlogged and well-watered conditions. Waterlogging during the onion bulb development stage irreversibly affects the bulb development process of sensitive genotypes. The recovered plants of the sensitive genotypes failed to produce bulbs of marketable size and quality. The crucial physiological processes governing onion bulb development are photosynthesis and carbon assimilate partitioning, which determine the bulb size and shape (Zhang et al., 2016). Bulb yield reduction in sensitive genotypes might be due to an imbalance between the sources and sink relationship under waterlogging conditions. Translocation of photosynthates from aerial parts of the plants to the bulbs was significantly affected under waterlogged conditions. Wang et al. (2019) report that plant growth and development were severely affected under waterlogged conditions due to decrease in the stomatal conductance, CO_2_ assimilation rate, photosynthesis, and nutrient imbalance, which ultimately reduced crop yield. By contrast, comparatively higher bulb yield of stress-tolerant genotypes might be due to high assimilate translocation to the developing bulbs that regulate the bulb size and yield. Thus, under stress conditions, the genotypes with better root architecture can maintain aerial growth with minimum leaf damage and optimum photosynthesis activity, thereby sustaining the bulb yield. Therefore, these traits can be used as criteria for selecting waterlogging-tolerant onion genotypes. The waterlogging-tolerant genotypes identified from the present study, namely, Acc. 1666, Acc. 1630, and W-355 and popular cultivar Bhima Dark Red, have been further evaluated on larger plots during monsoon season 2019–20 and 2020–21 at ICAR-Directorate of Onion and Garlic Research, Pune, India, for confirmation. These genotypes show better crop stand under waterlogged conditions and produced bulb yield comparable to normal conditions (Supplementary File 2). In the study on wheat, the association of yield-contributing traits with grain yield under waterlogging conditions was used as the criteria for selecting tolerant and stable genotypes, which supports our findings (Singh et al., 2018). The genotypes with better plant growth and bulb yield under waterlogging conditions can be employed in breeding programs for developing waterlogging-tolerant onion varieties.

In the present study, PCA analysis revealed that the number of photosynthetically active leaves, LA, LL, total chlorophyll, MSI, and bulb yield could be used for evaluating criteria for the performance of contrasting onion genotypes. The genotypes with a high greenness index and large number of photosynthetically active leaves can assimilate more carbon with maximum translocation toward the bulbs, thereby producing higher bulb yield under waterlogging conditions. According to the study, traits with a coefficient value > 0.3 have a greater effect than those with a coefficient value < 0.3 (Raji, 2002). As per this criterion, the three PC groups in the current study accounted for 76.74% of the total variation under waterlogging stress, indicating the structure underlying the parameters analyzed. The highest coefficient in the PC 1 group was observed for bulb yield, LA, and the number of leaves, indicating that these traits are the vital phenotypic traits that are directly linked with the photosynthesis ability of a particular genotype under stress. The highest contribution of LA was used as a criterion for selecting flood-tolerant mung bean genotypes (Kumar et al., 2013). Our previous study revealed the highest contribution of bulb yield for identifying drought-tolerant onion genotypes (Gedam et al., 2021). Sundgren et al. (2018) report similar findings in wheat and barley, with which waterlogging-tolerant and sensitive genotypes were identified depending upon the phenotypic and yield traits. Overall, our results indicate that the PCA analysis is a systematic approach to simultaneously screen several onion genotypes according to the variations in their phenotypic, physiological, biochemical, and yield traits under waterlogging stress. In our previous study, diverse onion genotypes were grouped into five clusters using PCA that helped in identifying the drought-tolerant onion genotypes (Gedam et al., 2021). This method of classification for various genotypes is used for other crops under different environmental conditions (Islam et al., 2007; Ghodke et al., 2019; Kołton et al., 2020).

## Conclusion

Our results show that plant survival and bulb yield traits under waterlogged conditions can be used for selecting sensitive and tolerant genotypes. The present study also highlights the unique genetic diversity among the onion genotypes for waterlogging tolerance. Genotype Acc. 1666 was identified as waterlogging-tolerant and W-344 was identified as the most sensitive onion genotype among the 100 genotypes selected in this study. The identified contrasting genotypes can be employed in molecular marker–assisted breeding to identify the waterlogging-tolerance genes or markers. In addition, genotype Acc. 1666 can be selected for cultivation in the monsoon season as a waterlogging-tolerant variety. Furthermore, the introduction of tolerance traits from this genotype to the high-yield onion cultivars could be used as a source for improving their bulb yield under waterlogged conditions.

## Data Availability Statement

The original contributions presented in the study are included in the article/Supplementary Material, further inquiries can be directed to the corresponding author/s.

## Author Contributions

PG: project administration, resources, supervision, methodology, analysis, and writing—original draft. DS: methodology, biochemical analysis, statistical analysis, writing—original draft, review, and editing. TA: project administration, resources, supervision, analysis, writing—original draft, review, and editing. SG: statistical analysis and writing—original draft. SJG: resources, supervision, writing—original draft, review, and editing. VM and AG: supervision and resources. MS: project administration, resources, and supervision. All authors listed have made substantial, direct, and intellectual contribution to the work.

## Conflict of Interest

The authors declare that the research was conducted in the absence of any commercial or financial relationships that could be construed as a potential conflict of interest.

## Publisher’s Note

All claims expressed in this article are solely those of the authors and do not necessarily represent those of their affiliated organizations, or those of the publisher, the editors and the reviewers. Any product that may be evaluated in this article, or claim that may be made by its manufacturer, is not guaranteed or endorsed by the publisher.
