# Supplementary figures and images for: Screening of Onion (Allium cepa L.) Genotypes for Waterlogging Tolerance
Source: Front Plant Sci. 2022 Jan 5;12:727262. doi: 10.3389/fpls.2021.727262 (PMC8766973; doi:10.3389/fpls.2021.727262)

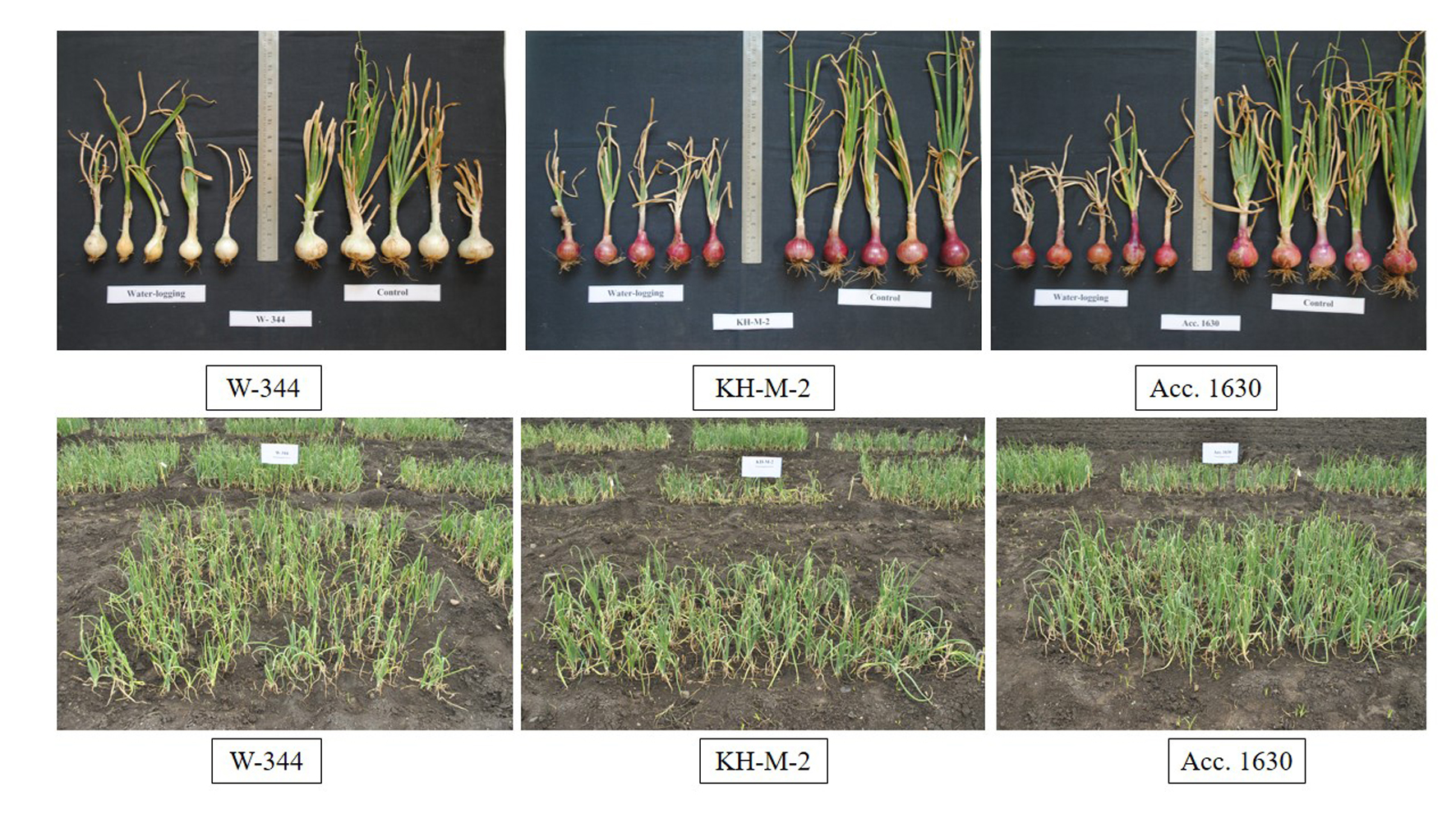

Supplement: Supplementary file 2 [file Image_1.JPEG]

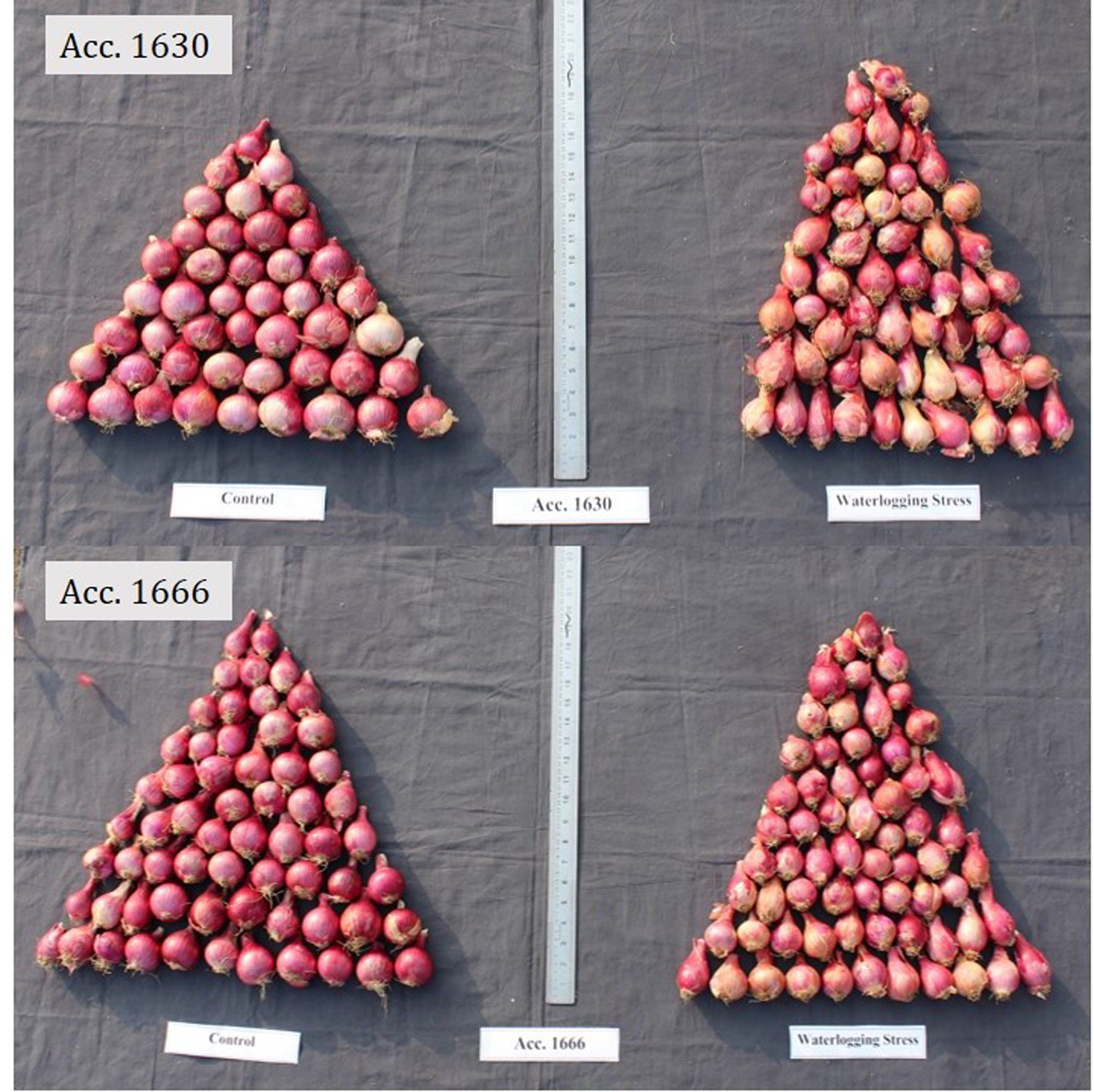

Supplement: Supplementary file 3 [file Image_2.JPEG]
