# Supplementary material for: Screening of Onion (Allium cepa L.) Genotypes for Waterlogging Tolerance
Source: Front Plant Sci. 2022 Jan 5;12:727262. doi: 10.3389/fpls.2021.727262 (PMC8766973; doi:10.3389/fpls.2021.727262)
Supplement: Supplementary file 1 [file Data_Sheet_1.zip › Sup/Table 1.DOC]

| **Year** | **Month** | **Average Temp. (Max)** | **Average Temp. (Min)** | **Average Relative Humidity (Max)** | **Average Relative Humidity (Min)** | **Average Velocity (mm )** | **Average Sunshine (Hr)** | **Total Rainfall (mm)** | **Average Evaporation** |
| --- | --- | --- | --- | --- | --- | --- | --- | --- | --- |
| 2017 | June | 32.6 | 22.3 | 82.9 | 62.1 | 10.2 | 6.7 | 209.0 | 3.9 |
|  | July | 28.0 | 20.6 | 89.9 | 80.6 | 12.8 | 3.7 | 269.2 | 0.9 |
|  | August | 27.7 | 18.7 | 89.0 | 79.4 | 6.0 | 2.7 | 231.4 | 1.3 |
|  | September | 30.9 | 19.2 | 87.0 | 65.9 | 3.0 | 5.4 | 91.2 | 2.0 |
|  | October | 31.2 | 17.4 | 80.4 | 65.2 | 2.1 | 6.6 | 140.4 | 2.8 |
| 2018 | June | 32.0 | 19.5 | 84.9 | 68.7 | 9.1 | 4.1 | 109.0 | 4.0 |
|  | July | 27.0 | 18.4 | 87.4 | 84.9 | 12.4 | 2.4 | 161.4 | 1.0 |
|  | August | 27.0 | 18.8 | 91.1 | 82.9 | 9.8 | 2.3 | 72.4 | 1.5 |
|  | September | 31.2 | 17.3 | 77.1 | 63.6 | 5.5 | 6.8 | 19.0 | 3.6 |
|  | October | 34.4 | 16.5 | 79.2 | 60.5 | 3.4 | 8.6 | 110.0 | 3.2 |
| 2019 | June | 33.9 | 21.7 | 81.9 | 67.6 | 8.3 | 7.0 | 182.8 | 4.9 |
|  | July | 28.6 | 21.6 | 90.8 | 80.7 | 8.0 | 2.8 | 350.7 | 1.3 |
|  | August | 28.1 | 20.6 | 83.9 | 76.7 | 10.1 | 3.6 | 169.8 | 1.1 |
|  | September | 28.0 | 20.5 | 89.3 | 75.5 | 8.2 | 3.7 | 199.0 | 1.0 |
|  | October | 29.6 | 20.3 | 87.1 | 68.6 | 3.8 | 5.7 | 136.8 | 1.7 |

**Supplementary Table 1.** Monthly Metrological data recorded during the experiment period for three constitutive years (2017, 2018, and 2019)
