# Supplementary material for: Screening of Onion (Allium cepa L.) Genotypes for Waterlogging Tolerance
Source: Front Plant Sci. 2022 Jan 5;12:727262. doi: 10.3389/fpls.2021.727262 (PMC8766973; doi:10.3389/fpls.2021.727262)
Supplement: Supplementary file 1 [file Data_Sheet_1.zip › Sup/Table 2.DOC]

**Supplementary Table 2**. Grouping of 100 onion genotypes in three different clusters based on Euclidean distance .

| **Cluster** | **Number** | **Name of genotypes** |
| --- | --- | --- |
| **Cluster I** | 19 | W-208, W-355, W-395, W-043, W-172, Acc. 1133, Acc. 1615, Acc. 1619, Acc. 1622, Acc. 1653, Acc. 1661, Acc. 1664, Acc. 1666, Acc. 1706, Acc. 1708, DOGR Hy-3, DOGR Hy-50, KH-M-2, RGP-5 |
| **Cluster II** | 54 | W-448, W-361, W-408, W-085, W-396, W-302, W-507, W-453, W-344, W-517, W-306, Acc. 1604, Acc. 1608, Acc. 1609, Acc. 1616, Acc. 1618, Acc. 1620, Acc. 1623, Acc. 1625, Acc. 1626, Acc. 1627, Acc. 1628, Acc. 1635, Acc. 1636, Acc. 1637, Acc. 1638, Acc. 1639, Acc. 1644, Acc. 1649, Acc. 1651, Acc. 1658, Acc. 1667, Acc. 1697, Acc. 1702, Acc. 1696, DOGR Hy-1, DOGR Hy-6, DOGR Hy-7, DOGR Hy-56, KH-M-1, KH-M-3, RGP-1, RGP-2, RGP-3, RGP-4, Bhima Shweta, Bhima Safed, Bhima Shubhra, Bhima Super, Bhima Red, Bhima Raj, Bhima Dark Red, Bhima Shakti, Phule Safed |
| **Cluster III** | 27 | W-504, Acc. 1606, Acc. 1613, Acc. 1617, Acc. 1621, Acc. 1624, Acc. 1629, Acc. 1630, Acc. 1632, Acc. 1633, Acc. 1640, Acc. 1645, Acc. 1656, Acc. 1660, Acc. 1663, Acc. 1668, Acc. 1701, Acc. 1703, Acc. 1709, Acc. 1712, Acc. 1704, Acc. 1714, DOGR Hy-2, DOGR Hy-4, DOGR Hy-8, KH-M-4, Bhima Kiran |
