# Supplementary material for: Screening of Onion (Allium cepa L.) Genotypes for Waterlogging Tolerance
Source: Front Plant Sci. 2022 Jan 5;12:727262. doi: 10.3389/fpls.2021.727262 (PMC8766973; doi:10.3389/fpls.2021.727262)
Supplement: Supplementary file 1 [file Data_Sheet_1.zip › Sup/Table 3.DOC]

**Supplementary Table 3. Three-way ANOVA for morphological, physiological, biochemical, and yield traits under well-watered and waterlogging condition**

| Source | DF | Mean Square | F-Ratio | p-Value | LSD | Significant |
| --- | --- | --- | --- | --- | --- | --- |
| **Plant height (PH)** | | | | | | |
| rep | 2 | 1.08 | 0.26 | 0.7952 | - | NS |
| year | 1 | 172.19 | 40.99 | 0.0235 | 1.47 | * |
| wl | 1 | 948.31 | 152.93 | 0.0002 | 1.15 | *** |
| year*wl | 1 | 14.07 | 2.27 | 0.2066 | NS | NS |
| gy | 11 | 127.12 | 15.36 | <.0001 | 2.33 | *** |
| year*gy | 11 | 62.77 | 7.59 | <.0001 | 3.30 | *** |
| wl*gy | 11 | 12.08 | 1.46 | 0.1613 | NS | NS |
| year*wl*gy | 11 | 5.83 | 0.7038 | 0.7317 | NS | NS |
| **Number of leaves (NL)** | | | | | | |
| rep | 2 | 0.37 | 0.85 | 0.5406 | - | NS |
| year | 1 | 59.21 | 137.26 | 0.0072 | 0.47 | ** |
| wl | 1 | 246.32 | 1232.53 | <.0001 | 0.21 | *** |
| year*wl | 1 | 2.87 | 14.37 | 0.0193 | 0.51 | * |
| gy | 11 | 4.38 | 10.38 | <.0001 | 0.53 | *** |
| year*gy | 11 | 2.29 | 5.44 | <.0001 | 0.74 | *** |
| wl*gy | 11 | 3.75 | 8.89 | <.0001 | 0.74 | *** |
| year*wl*gy | 11 | 1.09 | 2.58 | 0.007 | 1.05 | ** |
| **Leaf length (LL)** | | | | | | |
| rep | 2 | 3.26 | 0.77 | 0.5663 | - | NS |
| year | 1 | 14.87 | 3.49 | 0.2026 | NS | NS |
| wl | 1 | 926.24 | 259.17 | <.0001 | 0.87 | *** |
| year*wl | 1 | 20.71 | 5.80 | 0.0738 | NS | NS |
| gy | 11 | 40.08 | 6.42 | <.0001 | 2.03 | *** |
| year*gy | 11 | 72.33 | 11.58 | <.0001 | 3.02 | *** |
| wl*gy | 11 | 38.25 | 6.12 | <.0001 | 2.87 | *** |
| year*wl*gy | 11 | 37.73 | 6.04 | <.0001 | 4.05 | *** |
| **Leaf area (LA)** | | | | | | |
| rep | 2 | 5.05 | 1.01 | 0.4978 | - | NS |
| year | 1 | 446.69 | 89.28 | 0.011 | 1.60 | * |
| wl | 1 | 1107.85 | 1218.42 | <.0001 | 0.44 | *** |
| year*wl | 1 | 1.60 | 1.76 | 0.2555 | NS | NS |
| gy | 11 | 56.68 | 17.85 | <.0001 | 1.45 | *** |
| year*gy | 11 | 22.91 | 7.22 | <.0001 | 2.40 | *** |
| wl*gy | 11 | 18.09 | 5.70 | <.0001 | 2.04 | *** |
| year*wl*gy | 11 | 3.65 | 1.15 | 0.3332 | NS | NS |
| **Shoot length/Root length (RL/SL)** | | | | | | |
| rep | 2 | 0.0007 | 23.68 | 0.0405 | - | * |
| year | 1 | 0.05 | 1751.43 | 0.0006 | 0.00 | ** |
| wl | 1 | 0.043 | 67.05 | 0.0012 | 0.01 | * |
| year*wl | 1 | 0.013 | 20.24 | 0.0108 | 0.02 | * |
| gy | 11 | 0.035 | 24.58 | <.0001 | 0.03 | *** |
| year*gy | 11 | 0.005 | 3.14 | 0.0013 | 0.04 | ** |
| wl*gy | 11 | 0.021 | 14.65 | <.0001 | 0.04 | *** |
| year*wl*gy | 11 | 0.009 | 6.59 | <.0001 | 0.06 | *** |
| **Membrane stability index (MSI)** | | | | | | |
| rep | 2 | 5.86 | 3.74 | 0.2109 | - | NS |
| year | 1 | 0.04 | 0.02 | 0.8917 | NS | NS |
| wl | 1 | 7117.10 | 19879.65 | <.0001 | 0.28 | *** |
| year*wl | 1 | 65.35 | 182.54 | 0.0002 | 0.93 | ** |
| gy | 11 | 119.03 | 40.24 | <.0001 | 1.40 | *** |
| year*gy | 11 | 39.26 | 13.27 | <.0001 | 1.97 | *** |
| wl*gy | 11 | 71.40 | 24.14 | <.0001 | 1.91 | *** |
| year*wl*gy | 11 | 18.97 | 6.41 | <.0001 | 2.79 | *** |
| **Chlorophyll** | | | | | | |
| rep | 2 | 0.01 | 0.39 | 0.7174 | - | NS |
| year | 1 | 18.24 | 1875.75 | 0.0005 | 0.07 | *** |
| wl | 1 | 44.74 | 1805.58 | <.0001 | 0.07 | *** |
| year*wl | 1 | 0.24 | 9.75 | 0.0354 | 0.10 | * |
| gy | 11 | 3.75 | 36.34 | <.0001 | 0.26 | *** |
| year*gy | 11 | 2.29 | 22.23 | <.0001 | 0.37 | *** |
| wl*gy | 11 | 0.71 | 6.87 | <.0001 | 0.37 | *** |
| year*wl*gy | 11 | 0.37 | 3.61 | 0.0003 | 0.51 | ** |
| **Yield** | | | | | | |
| rep | 2 | 2.51 | 0.59 | 0.6294 | - | NS |
| year | 1 | 89.68 | 21.04 | 0.0444 | 1.48 | * |
| wl | 1 | 5436.60 | 1707.11 | <.0001 | 0.83 | *** |
| year*wl | 1 | 28.20 | 8.85 | 0.0409 | 1.17 | * |
| gy | 11 | 97.69 | 32.81 | <.0001 | 1.40 | *** |
| year*gy | 11 | 21.70 | 7.29 | <.0001 | 1.98 | *** |
| wl*gy | 11 | 37.19 | 12.49 | <.0001 | 2.29 | *** |
| year*wl*gy | 11 | 4.96 | 1.66 | 0.0951 | NS | NS |
| **Total soluble solids (TSS)** | | | | | | |
| rep | 2 | 0.02 | 2.78 | 0.2643 | - | NS |
| year | 1 | 5.22 | 654.85 | 0.0015 | 0.06 | ** |
| wl | 1 | 114.10 | 5987.36 | <.0001 | 0.06 | *** |
| year*wl | 1 | 5.28 | 277.19 | <.0001 | 2.78 | *** |
| gy | 11 | 2.23 | 24.01 | <.0001 | 0.25 | *** |
| year*gy | 11 | 1.00 | 10.74 | <.0001 | 2.01 | *** |
| wl*gy | 11 | 1.09 | 11.66 | <.0001 | 2.00 | *** |
| year*wl*gy | 11 | 1.28 | 13.70 | <.0001 | 2.00 | *** |
| **Phenol** | | | | | | |
| rep | 2 | 0.58 | 3.69 | 0.2132 | - | NS |
| year | 1 | 86.86 | 550.44 | 0.0018 | 0.28 | ** |
| wl | 1 | 14.67 | 39.30 | 0.0033 | 0.28 | ** |
| year*wl | 1 | 0.87 | 2.32 | 0.2026 | NS | NS |
| gy | 11 | 2.72 | 14.85 | <.0001 | 0.35 | *** |
| year*gy | 11 | 5.68 | 30.98 | <.0001 | 0.53 | *** |
| wl*gy | 11 | 0.31 | 1.69 | 0.0884 | NS | NS |
| year*wl*gy | 11 | 0.39 | 2.13 | 0.0258 | 0.69 | * |
| **Pyruvic acid** | | | | | | |
| rep | 2 | 0.03 | 0.70 | 0.5898 | - | NS |
| year | 1 | 1.04 | 24.65 | 0.0383 | 0.15 | * |
| wl | 1 | 5.80 | 679.18 | <.0001 | 0.04 | *** |
| year*wl | 1 | 0.17 | 19.46 | 0.0116 | 0.06 | * |
| gy | 11 | 0.66 | 21.50 | <.0001 | 0.14 | *** |
| year*gy | 11 | 1.52 | 49.14 | <.0001 | 0.20 | *** |
| wl*gy | 11 | 0.38 | 12.31 | <.0001 | 0.20 | *** |
| year*wl*gy | 11 | 0.43 | 13.85 | <.0001 | 1.99 | *** |
| **Antioxidant activity (AOX)** | | | | | | |
| rep | 2 | 0.14 | 5.45 | 0.155 | - | NS |
| year | 1 | 3.04 | 117.27 | 0.0084 | 0.12 | ** |
| wl | 1 | 5.58 | 145.51 | 0.0003 | 0.09 | ** |
| year*wl | 1 | 0.01 | 0.38 | 0.5728 | NS | NS |
| gy | 11 | 1.11 | 14.89 | <.0001 | 0.22 | *** |
| year*gy | 11 | 1.27 | 17.21 | <.0001 | 0.31 | *** |
| wl*gy | 11 | 0.19 | 2.50 | 0.0089 | 0.31 | ** |
| year*wl*gy | 11 | 0.48 | 6.50 | <.0001 | 0.44 | *** |

* Indicates significance at the 0.05 level of significance, ** Indicates significance at the 0.01 level of significance, ** Indicates significance at the <0.0001 level of significance, LSD-Lease significant difference
